# Metabolic radiolabeling and in vivo PET imaging of cytotoxic T lymphocytes to guide combination adoptive cell transfer cancer therapy

**DOI:** 10.1186/s12951-021-00924-2

**Published:** 2021-06-10

**Authors:** Dehua Lu, Yanpu Wang, Ting Zhang, Feng Wang, Kui Li, Shixin Zhou, Hua Zhu, Zhi Yang, Zhaofei Liu

**Affiliations:** 1 Medical Isotopes Research Center and Department of Radiation Medicine, School of Basic Medical Sciences, Peking University Health Science Center, Beijing, 100191 China; 2 Key Laboratory of Carcinogenesis and Translational Research (Ministry of Education/Beijing), Department of Nuclear Medicine, Peking University Cancer Hospital & Institute, Beijing, 100142 China; 3 Department of Cell Biology, School of Basic Medical Sciences, Peking University Health Science Center, Beijing, 100191 China; 4 NMPA Key Laboratory for Research and Evaluation of Radiopharmaceuticals (National Medical Products Administration), Peking University Cancer Hospital & Institute, Beijing, 100142 China

**Keywords:** Positron emission tomography, Radiolabeling, Adoptive cell transfer, Focal adhesion kinase inhibition, Image-guided therapy

## Abstract

**Background:**

Adoptive T cell transfer-based immunotherapy yields unsatisfactory results in the treatment of solid tumors, partially owing to limited tumor infiltration and the immunosuppressive microenvironment in solid tumors. Therefore, strategies for the noninvasive tracking of adoptive T cells are critical for monitoring tumor infiltration and for guiding the development of novel combination therapies.

**Methods:**

We developed a radiolabeling method for cytotoxic T lymphocytes (CTLs) that comprises metabolically labeling the cell surface glycans with azidosugars and then covalently conjugating them with ^64^Cu-1,4,7-triazacyclononanetriacetic acid-dibenzo-cyclooctyne (^64^Cu-NOTA-DBCO) using bioorthogonal chemistry. ^64^Cu-labeled control-CTLs and ovalbumin-specific CTLs (OVA-CTLs) were tracked using positron emission tomography (PET) in B16-OVA tumor-bearing mice. We also investigated the effects of focal adhesion kinase (FAK) inhibition on the antitumor efficacy of OVA-CTLs using a poly(lactic-*co*-glycolic) acid (PLGA)-encapsulated nanodrug (PLGA-FAKi).

**Results:**

CTLs can be stably radiolabeled with ^64^Cu with a minimal effect on cell viability. PET imaging of ^64^Cu-OVA-CTLs enables noninvasive mapping of their in vivo behavior. Moreover, ^64^Cu-OVA-CTLs PET imaging revealed that PLGA-FAKi induced a significant increase in OVA-CTL infiltration into tumors, suggesting the potential for a combined therapy comprising OVA-CTLs and PLGA-FAKi. Further combination therapy studies confirmed that the PLGA-FAKi nanodrug markedly improved the antitumor effects of adoptive OVA-CTLs transfer by multiple mechanisms.

**Conclusion:**

These findings demonstrated that metabolic radiolabeling followed by PET imaging can be used to sensitively profile the early-stage migration and tumor-targeting efficiency of adoptive T cells in vivo. This strategy presents opportunities for predicting the efficacy of cell-based adoptive therapies and for guiding combination regimens.

**Graphic Abstract:**

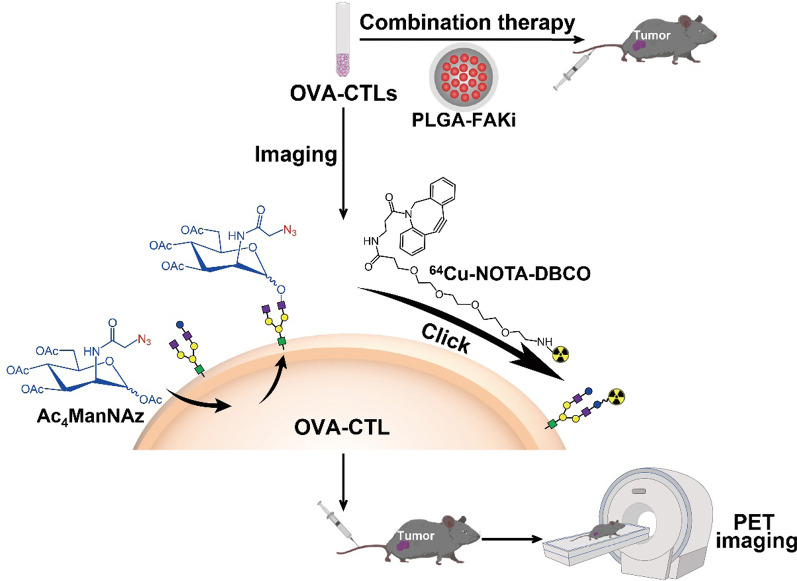

**Supplementary Information:**

The online version contains supplementary material available at 10.1186/s12951-021-00924-2.

## Introduction

Cell-based therapies, such as the adoptive cell transfer of engineered T cells and natural killer cells, represent promising strategies in cancer immunotherapy. An example of such a strategy is the successful use of CD19-specific chimeric antigen receptor T cells (CAR-T cells) in the treatment of patients with relapsed B-cell lymphoma and acute lymphoblastic leukemia [1, 2]. However, while successful in hematological cancers, the application of CAR-T cell therapy in solid tumors is generally disappointing [3, 4]. There are many obstacles that hinder the effective treatment of solid tumors using CAR-T cells, such as a limited choice of target antigens for solid tumors [5], barriers to effective T-cell infiltration into the tumor [6, 7], and the abundance of immunosuppressive cells in the tumor microenvironment [8, 9]. Therefore, strategies to overcome these obstacles, such as combination therapies, may be necessary to improve the efficacy of CAR-T cell therapy in solid tumors.

Focal adhesion kinase (FAK) is a non-receptor tyrosine kinase that exists at the integrin assembly site; it promotes cellular signal transduction through extracellular matrix protein interactions [10]. FAK increases tumor cell proliferation by multiple mechanisms [11–13], and FAK inhibitors can regulate the tumor microenvironment to reduce fibrosis and promote immune surveillance [14]. Recently, FAK inhibition was reported to overcome the immunosuppressive tumor microenvironment and increased cytotoxic T cell infiltration [15]. These findings suggest that a combination therapy involving FAK inhibition may improve the efficacy of the adoptive cell transfer of effector T cells. However, many of the currently used FAK inhibitors are highly hydrophobic [16], thus limiting their clinical applications. Therefore, nanomedicine strategies, such as encapsulation with poly(lactic-co-glycolic) acid (PLGA) nanoparticles or liposomes which are composed by the United States Food and Drug Administration-approved agents, might expand the potential clinical translation of FAK inhibitors.

To better guide T cell-based cancer immunotherapy, spatial and temporal information about pharmacokinetics and biodistribution should be dynamically monitored in vivo following its administration [17]. Thus, the delivery dose of T cells can be optimized, effective combinations can be selected, and possible off-target side effects can be predicted. Currently, the clinically applied methods for monitoring CAR-T cells include serum profiling of the cytokines associated with T cell activation and examination of T cells in circulation or in the tumor using invasive biopsies. However, these methods are either limited in providing accurate whole-body information on the location of T cells, or are invasive procedures that cannot be repeated frequently [18]. Advanced molecular imaging techniques, such as positron emission tomography (PET) and single-photon emission computed tomography (SPECT), are now paving the way for noninvasive characterization and quantification of biological processes in living subjects [19]. Cutting-edge molecular imaging techniques would provide the potential for in vivo imaging of the dynamics of T cell transfer in a highly sensitive manner.

**Fig. 1:**
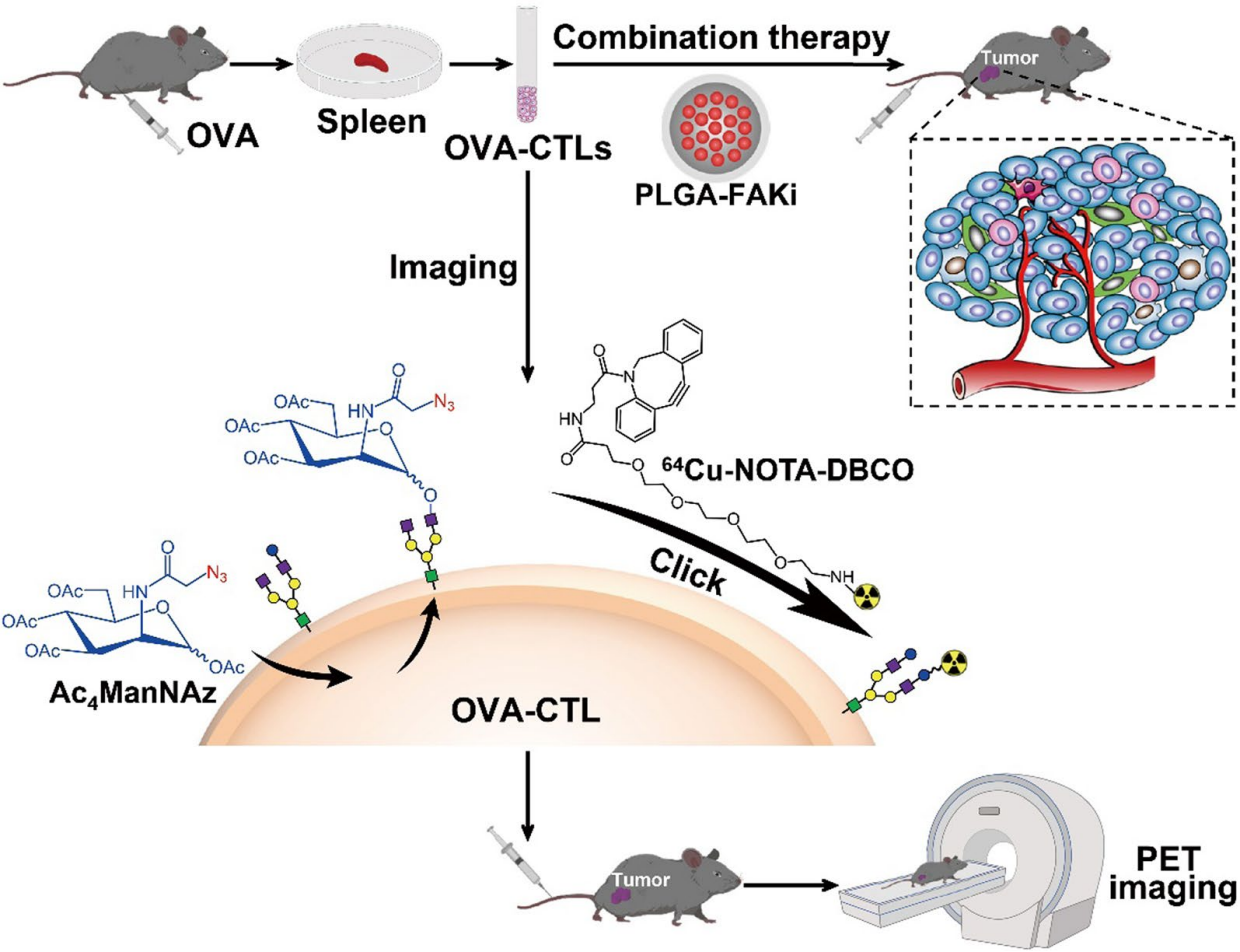
Schematic overview of OVA-specific cytotoxic T lymphocyte (OVA-CTL)-based tumor imaging and therapy. The OVA-CTLs were metabolically labeled with ^64^Cu, and injected into tumor-bearing mice for small-animal PET imaging. For the therapy studies, the OVA-CTLs were injected into tumor-bearing mice with the combination of a PLGA-FAKi nanodrug (*FAKi* focal adhesion kinase inhibitor)

Metabolic glycoengineering is a robust approach that exploits biosynthesis to incorporate reactive functional groups (e.g., azide-modified oligosaccharides) on the surfaces of cells [20, 21]. Metabolically glycoengineered cells can then be labeled by bioorthogonal click chemistry under physiological conditions without interfering with normal biological functions [22, 23]. In this study, we hypothesized that metabolic glycoengineering to express an azide group on the cell surface, followed by bioorthogonal chemistry could be used to radiolabel cytotoxic T lymphocytes (CTLs). We thus radiolabeled CTLs with ^64^Cu using this strategy and evaluated the in vivo behaviors of the resulting ^64^Cu-labeled CTLs (^64^Cu-CTLs) by noninvasive PET imaging. Because FAK inhibition potentially promotes the infiltration of T cells into tumors and enhance immune surveillance [15], we investigated whether FAK inhibition using a PLGA nanodrug could improve the therapy efficacy of CTL-based immunotherapy, and also assessed the feasibility of PET imaging to predict their combinational therapy efficacy (Fig. 1).

## Results

### Adoptive cell transfer of OVA-specific CTLs has an antitumor effect

To investigate the potential antitumor effects of the adoptive transfer of CTLs, we first determined the in vitro cytotoxic effect of CTLs on tumor cells. CTLs were isolated from mice pretreated with either phosphate-buffered saline (PBS) or OVA, and the dynamic interaction between CTLs and B16-OVA cells was investigated using fluorescence staining. Compared to the control-CTLs, the ovalbumin-specific CTLs (OVA-CTLs) were gradually recruited to the surroundings of the tumor cells and induced marked cytolysis (Additional file 1: Figure S1a). Moreover, the co-culture of OVA-CTLs with B16-OVA cells led to a notable increase in tumor cell death, as determined by phosphatidyl inositol (PI) staining (Additional file 1: Figure S1b).

**Fig. 2:**
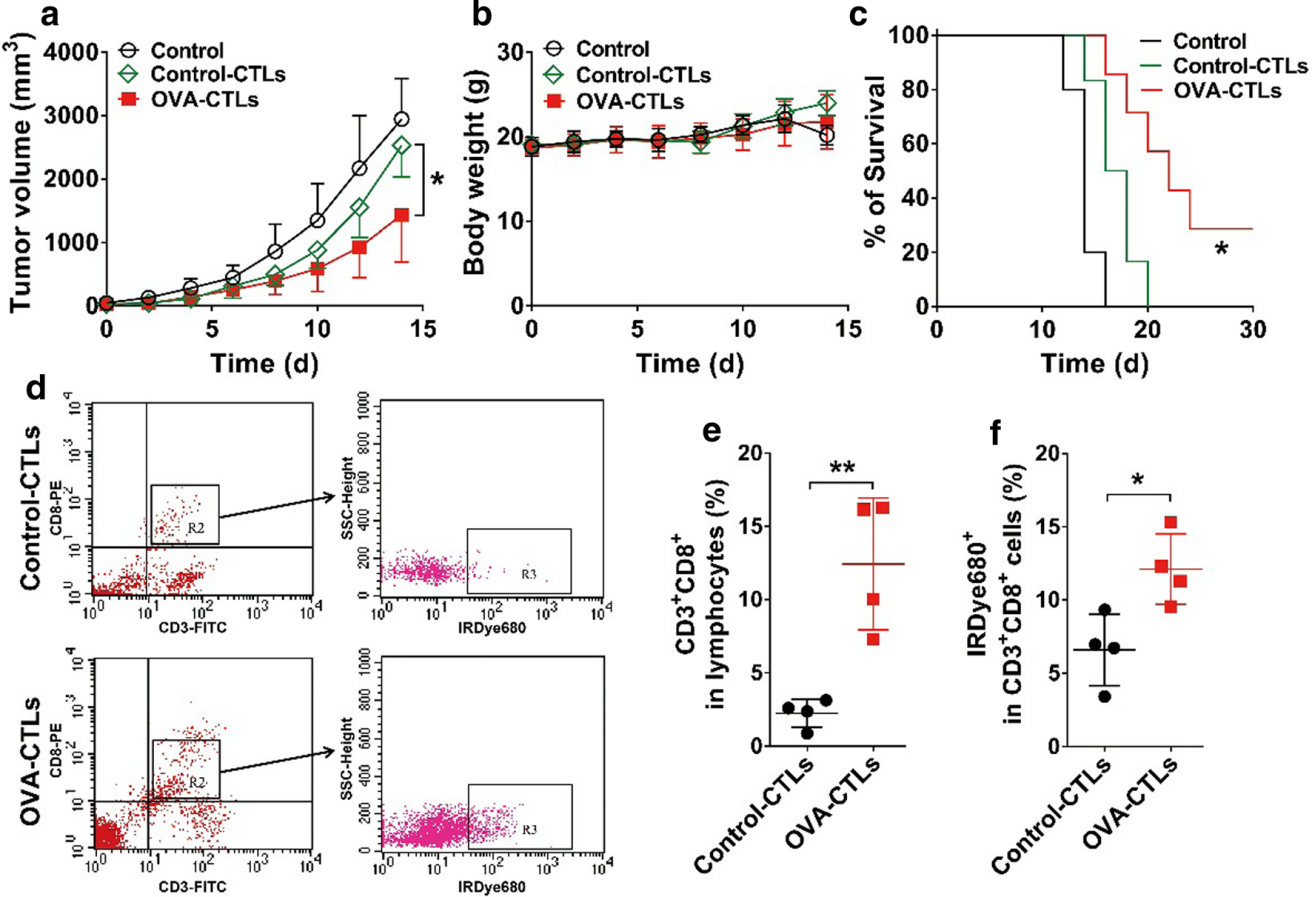
Antitumor effect and tumor accumulation of adoptive OVA-specific cytotoxic T lymphocytes (OVA-CTLs) in B16-OVA tumor-bearing C57BL/6 mice. **a**–**c** Tumor growth curves (**a**), body weights (**b**), and survival curves (**c**) of B16-OVA tumor-bearing C57BL/6 mice after intravenous injection of PBS (n = 5), control-CTLs (5 × 10^5^ cells every day for 4 days; n = 6), or OVA-CTLs (5 × 10^5^ cells every day for 4 days; n = 6). (**d**–**f**) Representative dot plots (**d**), quantification of CD8^+^ T cells (CD3^+^CD8^+^) (**e**), and the percentage of IRDye680-labled CTLs in CD3^+^CD8^+^ T cells (**f**) determined using flow cytometric analysis of single-cell suspensions derived from B16-OVA tumors harvested from C57BL/6 mice (n = 4/group) after the injection of IRDye680-labeled control-CTLs or IRDye680-labeled OVA-CTLs. Data are presented as mean ± SD. *, *P* < 0.05; **, *P* < 0.01

Next, we investigated the in vivo antitumor efficacy of OVA-CTLs in the B16-OVA tumor-bearing mouse model. As shown in Fig. 2a, compared to the control group, both the control-CTLs and OVA-CTLs led to tumor growth inhibition. The adoptive OVA-CTLs group exerted a significantly higher tumor growth inhibition effect than the control-CTLs group on day 14 (*P* < 0.05). There was no evident body weight loss in any of the groups **(**Fig. 2b), suggesting that adoptive CTL therapy is well tolerated in mice. The survival curve showed that treatment with OVA-CTLs led to improved mouse survival compared to the survival of the control and control-CTLs groups (*P* < 0.05, Fig. 2c). These results demonstrated the superior antitumor effects of the OVA-specific CTLs compared to the control-CTLs in the treatment of B16-OVA tumors.

To validate the specific accumulation of OVA-CTLs in the B16-OVA tumors, we prepared IRDye680-labeled CTLs and used fluorescence-activated cell sorting (FACS) to examine the tumor-infiltrated CTLs. In vitro cell binding studies confirmed that IRDye680-DBCO can easily conjugate to the surface of CTLs pretreated with Ac_4_ManNAz (Additional file 1: Figure S2a). We then administered IRDye680-labeled CTLs to B16-OVA tumor-bearing mice and analyzed single-cell suspensions obtained from them using FACS. As shown in Fig. 2d, e, there was a significantly higher proportion of CD3^+^CD8^+^ T cells in the OVA-CTLs group than in the control-CTL group (12.43 ± 2.26% vs. 2.25 ± 0.48%, *P* < 0.01, Fig. 2e). Among the sorted CD3^+^CD8^+^ T cells, there was a significantly higher proportion of IRDye680-labeled CTLs in the OVA-CTLs group than in the control-CTLs group (12.10 ± 1.21% vs. 6.60 ± 1.21%, *P* < 0.05, Fig. 2f). These results suggest that the OVA-specific CTLs were better able to accumulate in the tumors than the control-CTLs, which may explain why the OVA-CTLs had a significantly higher antitumor effect than the control-CTLs.

### Metabolic radiolabeling and PET imaging enable noninvasive tracking of CTLs

Because the extent of accumulation of CTLs in the tumor may be used to predict the antitumor efficacy of adoptive CTLs therapy, we next sought to develop a noninvasive PET imaging approach to track the in vivo tumor targeting of CTLs. To prepare radionuclide ^64^Cu-labeled CTLs, the CTLs were first pretreated with Ac_4_ManNAz to generate azide groups on their surfaces, and then labeled with ^64^Cu-NOTA-DBCO (Fig. 1). The efficiency, specificity, and effects on CTL function of this approach were then evaluated in vitro.

The recognition of specific antigens by T cells can induce interferon-γ (IFN-γ) secretion [24]. We thus examined IFN-γ expression levels to explore the interactions between CTLs and B16-OVA tumor cells and found that the OVA-CTLs produced significantly higher levels of IFN-γ than the control-CTLs (*P* < 0.0001, Additional file 1: Figure S2b). Moreover, pre-incubation with Ac_4_ManNAz, DBCO, or both Ac_4_ManNAz and DBCO produced similar IFN-γ expression levels to those produced by the OVA-CTLs (Additional file 1: Figure S2b), suggesting that incubation with Ac_4_ManNAz and subsequent ^64^Cu-NOTA-DBCO labeling had a negligible effect on CTL function.

**Fig. 3:**
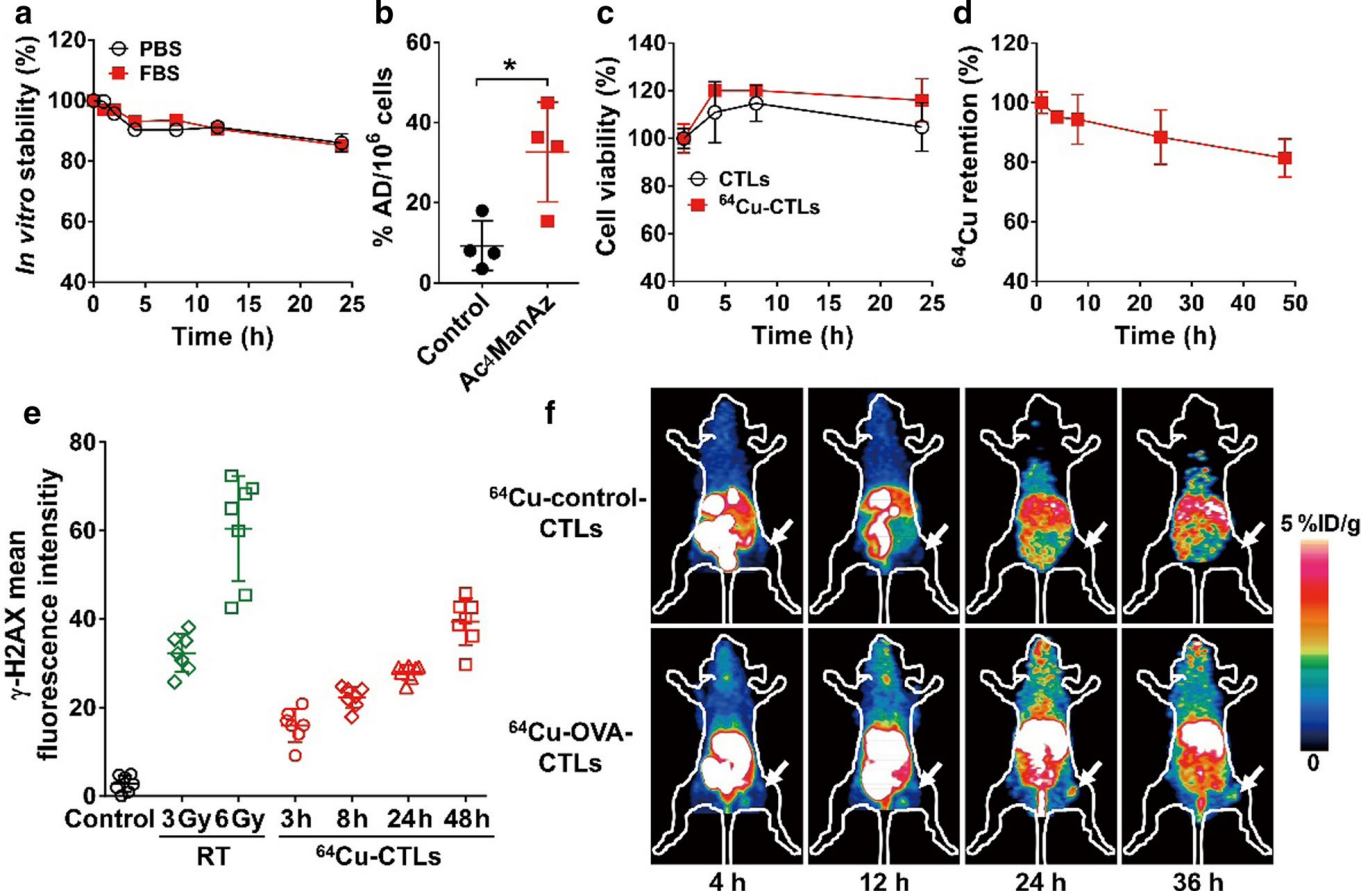
Metabolic ^64^Cu-radiolabeling of cytotoxic T lymphocytes (CTLs) and in vivo small-animal PET imaging. **a** In vitro stability of ^64^Cu-labeled NOTA-DBCO in PBS and FBS (n = 3). **b** Binding specificity of ^64^Cu-labeled NOTA-DBCO with Ac_4_ManNAz-incubated CTLs (n = 4). The results are expressed as percentage administered dose per million cells (%AD/10^6^ cells). **c** Cell viability of unlabeled CTLs or ^64^Cu-labeled CTLs (^64^Cu-CTLs) after culturing for various times (n = 5). **d** ^64^Cu retention on the surfaces of the CTLs after incubating in cell culture medium for various times (n = 4). **e** DNA damage of untreated CTLs (control), CTLs treated with 3 or 6 Gy of X-ray irradiation (RT), or ^64^Cu-labeled CTLs (3, 8, 24, and 48 h after labeling) as measured using γH2AX immunofluorescence staining (n = 7). **f** Small-animal PET images of ^64^Cu-labeled control-CTLs and ^64^Cu-labeled OVA-specific CTLs (OVA-CTLs) in B16-OVA tumor-bearing C57BL/6 mice at 4, 12, 24, and 36 h postinjection. Tumors are indicated by white arrows. Data are presented as mean ± SD. *, *P* < 0.05

DBCO was labeled with ^64^Cu using the chelator NOTA, and ^64^Cu-NOTA-DBCO exhibited favorable in vitro stability, with a radiochemical purity higher than 80% after 24 h in both PBS and fetal bovine serum (FBS) (Fig. 3a). The significantly higher binding of ^64^Cu-NOTA-DBCO to CTLs that had been pre-incubated with Ac_4_ManNAz than CTLs that had not been treated (32.66 ± 6.22% vs. 9.28 ± 3.08%, *P* < 0.05, Fig. 3b) indicated the specific binding of ^64^Cu-NOTA-DBCO on the Ac_4_ManNAz-treated CTLs.

Next, we determined the viability of CTLs after radiolabeling with ^64^Cu-NOTA-DBCO. The ^64^Cu-labeled CTLs had similar viability to the unlabeled CTLs up to 24 h (Fig. 3c), and the efflux of free ^64^Cu was less than 20% after 48 h (Fig. 3d). This indicated the low cytotoxicity of ^64^Cu labeling and the favorable stability of ^64^Cu-labeled CTLs. We also determined DNA damage in the radiolabeled CTLs using γH2AX staining. As shown in Fig. 3e, the DNA damage levels of ^64^Cu-labeled CTLs increased with time, and after 48 h reached similar levels to those in the CTLs exposed to 3 Gy of external X-ray radiation.

Then, the in vivo tracking of ^64^Cu-labeled CTLs was assessed using PET imaging. As shown in Fig. 3f, there were high levels of accumulation in the livers of both the mice injected with ^64^Cu-labeled OVA-CTLs and ^64^Cu-labeled control-CTLs. There was a marked increase in tumor accumulation in the B16-OVA tumor-bearing mice that had been injected with ^64^Cu-labeled OVA-CTLs compared to those that had been injected with ^64^Cu-labeled control-CTLs. To validate the accuracy of noninvasive PET imaging, ex vivo biodistribution studies were performed at 36 h postinjection. Consistent with the PET observations, the tumor uptake (1.76 ± 0.52%ID/g vs. 0.56 ± 0.12%ID/g, *P* < 0.05; Additional file 1: Figure S3a) and tumor-to-muscle ratios (1.79 ± 0.26 vs. 0.93 ± 0.17, *P* < 0.01; Additional file 1: Figure S3b) of the ^64^Cu-labeled OVA-CTLs were significantly higher than those of the ^64^Cu-labeled control-CTLs.

### PET imaging reveals increased tumor accumulation of OVA-CTLs in the tumors of mice treated with the FAKi nanodrug

**Fig. 4:**
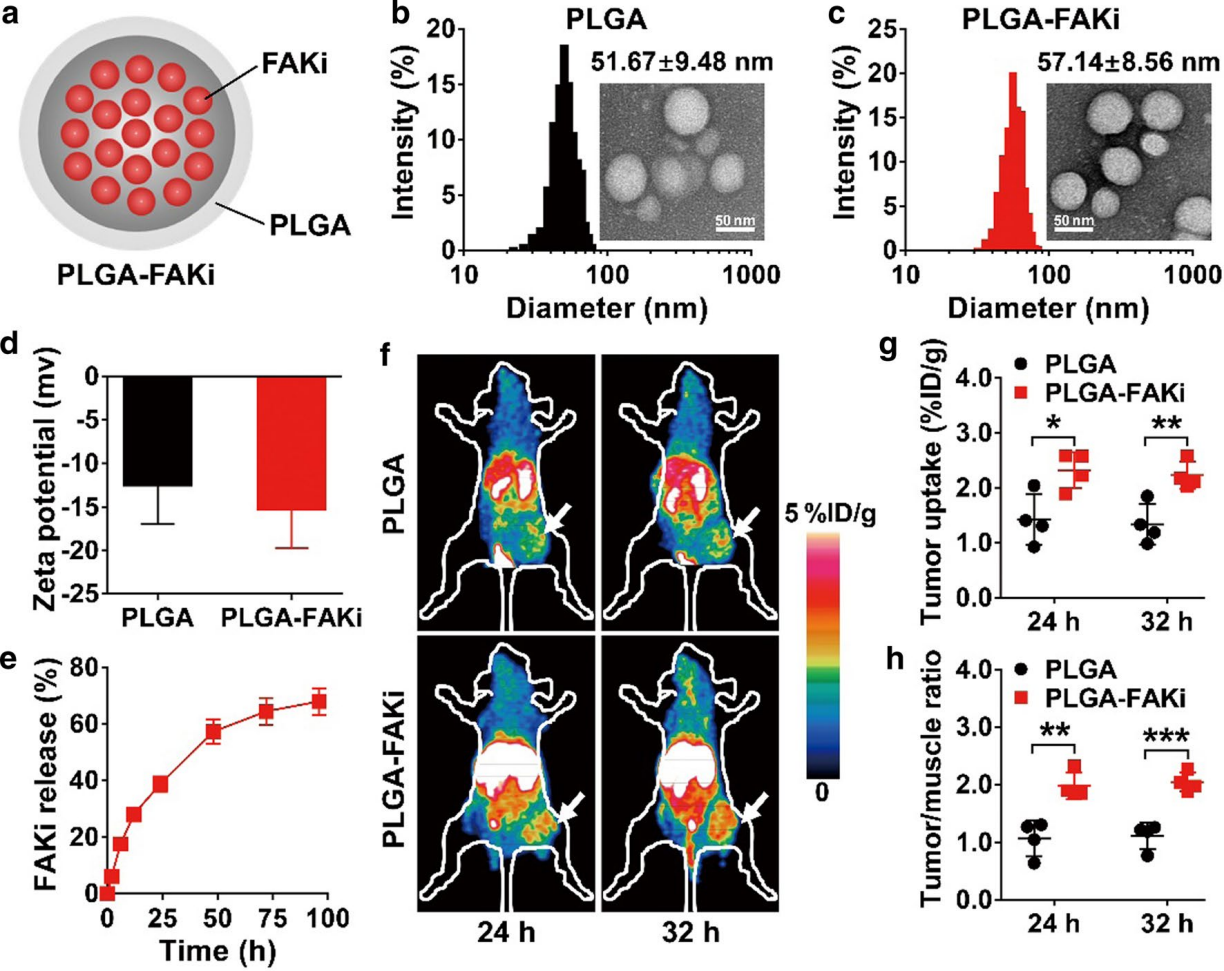
PET imaging of ^64^Cu-labeled OVA-specific cytotoxic T lymphocytes (OVA-CTLs) in B16-OVA tumor-bearing C57BL/6 mice treated with PLGA nanoparticles encapsulated with PF-562271 (PLGA-FAKi) or the control PLGA nanoparticles. **a** Schematic illustration of PLGA-FAKi. **b**, **c** Transmission electron microscope images and hydrodynamic diameters of PLGA (**b**) and PLGA-FAKi (**c**). **d** Surface zeta potentials of PLGA and PLGA-FAKi (n = 3). **e** Time-dependent profile of the release of PF-562271 (FAKi) from PLGA-FAKi nanoparticles dispersed in PBS at 37 °C (n = 3). **f**–**h** Small-animal PET images (**f**), quantified tumor uptake (**g**), and quantified tumor-to-muscle ratios (**h**) of ^64^Cu-OVA-CTLs at 24 and 32 h postinjection in B16-OVA tumor-bearing C57BL/6 mice after treatment with the control PLGA or PLGA-FAKi (n = 4/group). Tumors are indicated by white arrows. Data are presented as mean ± SD. *, *P* < 0.05; **, *P* < 0.01; ***, *P* < 0.001

Given the unsatisfactory antitumor effect of OVA-CTLs alone, we sought to develop a combination strategy to improve their efficacy. FAK is one of the major regulators of the tumor microenvironment, and its inhibition helps overcome tumor fibrotic and immunosuppressive microenvironments [15], suggesting that FAK inhibition may be used to increase the tumor infiltration of CTLs. However, small-molecule FAK inhibitors exhibit unfavorable in vivo pharmacokinetics. To this end, we encapsulated the FAK inhibitor PF-562271 with the PLGA nanoparticles (Fig. 4a). The final nanodrug named PLGA-FAKi had an average particle size of 57.14 ± 8.56 nm, which is slightly larger than that of the PLGA particles alone (51.67 ± 9.48) (Fig. 4b, c). According to the results of dynamic light scattering, the zeta potentials of PLGA and PLGA-FAKi were − 12.5 ± 4.5 mV and − 15.2 ± 4.6 mV, respectively (Fig. 4d). The cumulative release of PF-562271 from the PLGA-FAKi nanoparticles was determined to be over 60% at 96 h (Fig. 4e).

Next, we performed PET imaging of ^64^Cu-labeled OVA-CTLs to determine whether treatment with PLGA-FAKi increases the accumulation of OVA-CTLs in tumors. As shown in Fig. 4f, there was relatively little accumulation of the ^64^Cu-labeled OVA-CTLs in the tumors. In contrast, the tumors in the PLGA-FAKi treatment group were clearly visible after the injection of ^64^Cu-labeled OVA-CTLs, and there was high contrast with the contralateral background at both 24 and 32 h postinjection. The quantified tumor uptake values (%ID/g) and tumor-to-muscle ratios of the ^64^Cu-labeled OVA-CTLs in the PLGA-FAKi group were significantly higher than those in the PLGA control group at both 24 and 32 h postinjection (Fig. 4g, h). These results demonstrate that PLGA-FAKi treatment led to a marked increase in the accumulation of OVA-CTLs in the tumors of the B16-OVA tumor model.

### The FAKi nanodrug improves the antitumor efficacy of adoptive OVA-CTLs by modeling the tumor microenvironment

**Fig. 5:**
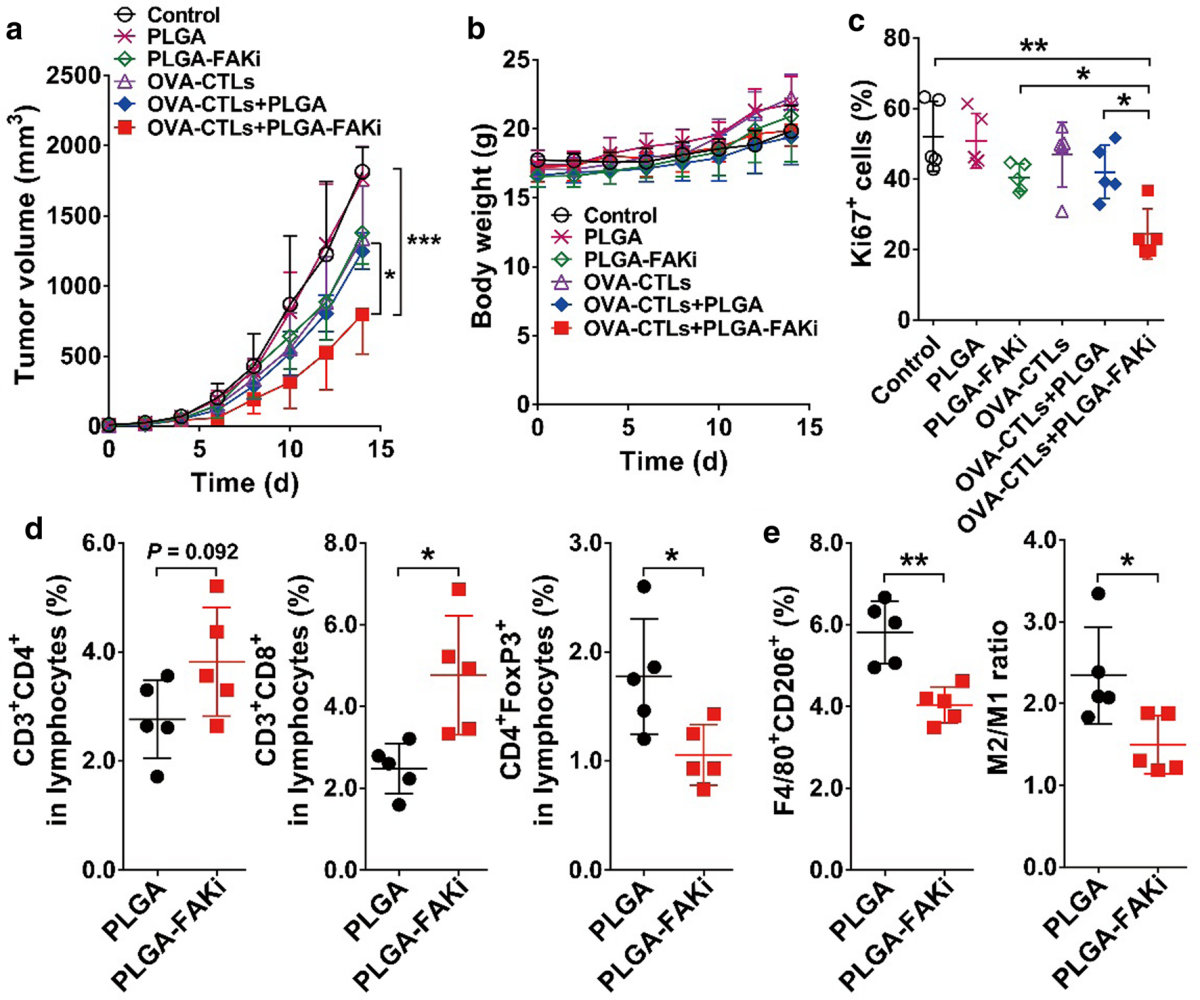
Combination therapy comprising adoptive transfer OVA-specific cytotoxic T lymphocytes (OVA-CTLs) and PLGA nanoparticles encapsulated with PF-562271 (PLGA-FAKi) in B16-OVA tumor-bearing C57BL/6 mice. **a**, **b** Tumor growth curves (**a**) and body weights (**b**) of B16-OVA tumor-bearing C57BL/6 mice (n = 7/group) after intravenous injection of PBS (control), PLGA, PLGA-FAKi, OVA-CTLs, OVA-CTLs plus PLGA, or OVA-CTLs plus PLGA-FAKi. **c** Quantitative analysis of Ki67-positive cells in the tumors harvested from mice after the indicated treatments (n = 5). **d**, **e** Quantified CD4^+^ T cells (CD3^+^CD4^+^), CD8^+^ T cells (CD3^+^CD8^+^), and T-regulatory cells (CD4^+^FoxP3^+^), and quantified M2 macrophages (F4/80^+^CD206^+^) and M2-to-M1 macrophage ratios in the B16-OVA tumors harvested from C57BL/6 mice (n = 5/group) after treatment with PLGA or PLGA-FAKi. Data are presented as mean ± SD. *, *P* < 0.05; **, *P* < 0.01; ***, *P* < 0.001

To validate the PET imaging results for the ^64^Cu-labeled OVA-CTLs, we investigated a combination therapy comprising adoptive CTLs and PLGA-FAKi. As shown in Fig. 5a, compared to the control group, PLGA alone did not have any effect on tumor growth suppression, but tumor growth was slightly inhibited by the treatments comprising PLGA-FAKi, OVA-CTLs, and OVA-CTLs plus PLGA. In contrast, treatment with PLGA-FAKi followed by adoptive OVA-CTL therapy had a more potent inhibitory effect on tumor growth than treatment with OVA-CTLs alone, PLGA-FAKi alone, or OVA-CTLs plus PLGA (*P* < 0.05). The body weights of the mice in the various groups did not change markedly (Fig. 5b), suggesting no obvious toxicity associated with the combination treatment of OVA-CTLs and PLGA-FAKi.

To further evaluate the effects of OVA-CTLs plus PLGA-FAKi on tumor cell proliferation, we stained the tumor tissues with Ki67. The tumors treated with OVA-CTLs plus PLGA-FAKi had significantly fewer proliferative cells than those treated with OVA-CTLs alone, PLGA-FAKi alone, or OVA-CTLs plus PLGA (*P* < 0.05; Additional file 1: Figure S4; Fig. 5c), which confirmed the improved antitumor proliferation effects of OVA-CTLs plus PLGA-FAKi.

Furthermore, we investigated the role of PLGA-FAKi in the infiltration of immune cells into the tumor microenvironment. As shown in Fig. 5d, there was a slight increase in the proportion of CD4^+^ T (CD3^+^CD4^+^) cells after PLGA-FAKi treatment, but the increase was not statistically significant. In contrast, there was a significant increase in the population percentage of CD8^+^ T (CD3^+^CD8^+^) cells, and a significant reduction in the population percentage of Treg cells (CD4^+^FoxP3^+^) within the tumor when the mice were treated with PLGA-FAKi (*P* < 0.05; Fig. 5d). Moreover, the percentages of M2 macrophages (F4/80^+^CD206^+^) and the M2/M1 ratios decreased significantly in the tumors treated with PLGA-FAKi compared to the corresponding values in those treated with PLGA alone (Fig. 5e). Taken together, these results demonstrate that treatment with PLGA-FAKi remodeled the immune inhibitory tumor microenvironment, thereby increasing the antitumor effect of the adoptive OVA-CTL therapy.

**Fig. 6:**
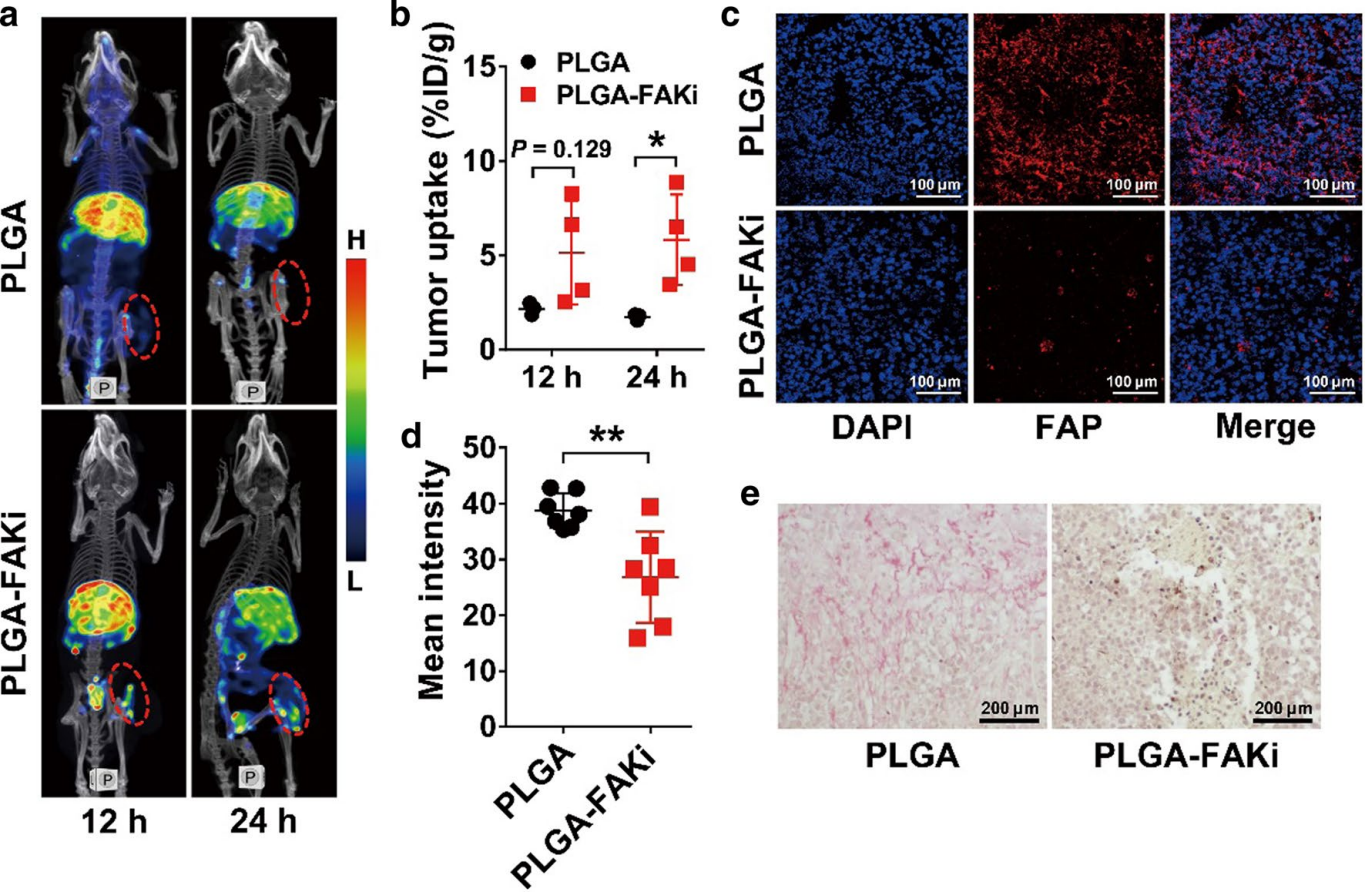
Effects of PLGA nanoparticle-encapsulated PF-562271 (PLGA-FAKi) treatment on the tumor microenvironment. **a**, **b** SPECT/CT images (**a**) and quantified tumor uptake (**b**) of ^99m^Tc-labeled human serum albumin in B16-OVA tumor-bearing C57BL/6 mice after treatment with PLGA (n = 3) or PLGA-FAKi (n = 4). Tumors are indicated by dashed circles. **c**–**e** Immunofluorescence staining (**c**) and quantified fluorescence intensity (**d**) of fibroblast activation protein (FAP), and Sirius red staining (**e**) in B16-OVA tumors harvested from C57BL/6 mice after treatment with PLGA or PLGA-FAKi (n = 7/group). Data are presented as mean ± SD. *, *P* < 0.05; **, *P* < 0.01

To further investigate the mechanisms of PLGA-FAKi with regard to the improved tumor suppression of OVA-CTLs, we performed SPECT imaging using ^99m^Tc-labeled human serum albumin (^99m^Tc-HSA) as a tumor perfusion marker. As shown in Fig. 6a, b, treatment with PLGA-FAKi led to a significantly increased tumor uptake of ^99m^Tc-HSA at 24 h, which was further confirmed by optical imaging using IRDye800-labeled HSA (Additional file 1: Figure S5a and S5b). This suggests that treatment with PLGA-FAKi increased the microvascular permeability of the tumors, thereby promoting the accumulation of OVA-CTLs. Moreover, the expression of fibroblast activation protein (FAP) was significantly reduced in the tumor tissues treated with PLGA-FAKi compared to the expression of FAP in those treated with PLGA (*P* < 0.01; Fig. 6c, d), and staining with Sirius Red also revealed a notable decrease in tumor fibrosis in the PLGA-FAKi-treated tumor sections (Fig. 6e).

## Discussion

As one of the dominating approaches for cancer immunotherapy, T cell-based adoptive transfer exhibits encouraging clinical results [25]. However, most of the current T cell-based therapies in the clinical practice are performed without knowing the in vivo distribution, tumor-targeting efficiency, and fate of the transferred cells [26]. Therefore, development of clinically available methods for noninvasive and quantitative characterization of the in vivo behaviors of adoptive transferred T cells is essential to optimize the therapy regimens and to predict the off-target toxicity. In this study, we established a strategy for metabolic radiolabeling and PET imaging of CTLs, and demonstrated its ability to track the early migration and guide the combination treatment regimens of CTL-based adoptive transfer therapy.

In vivo PET imaging of T cell behavior could be achieved by engineering cells to express reporter genes, such as those that encode herpes simplex virus type 1 thymidine kinase (HSV1-TK), somatostatin receptor subtype 2 (SSTR2), and prostate-specific membrane antigen (PSMA), and applying reporter-specific imaging probe pairs, i.e., ^18^F-FHBG [27, 28], ^68^ Ga-DOTATATE [29], and ^18^F-DCFPyL [30], respectively. However, the reporter gene strategies have limitations due to their ability to induce immunogenicity within the host, and the nonspecific uptake of the corresponding imaging probes can also be observed in vivo [31]. As an alternative method, we reported in this study that CTLs can be directly radiolabeled ex vivo using metabolic tagging and then be tracked in vivo using PET imaging. This strategy is straightforward, and enables the direct visualization of the location of adoptively transferred T cells.

Other ex vivo methods for T cell labeling reported previously include the use of ^111^In-oxine [32] or ^89^Zr-oxine [33, 34], the use of ^64^Cu-PTSM [35], and the treatment of the TCR-specific monoclonal antibody KJ1-26 with radionuclides to label cells via endocytosis [36]. Most recently, mesoporous silica nanoparticles containing ^68^ Ga or ^89^Zr have also been used to label cells, enabling the tracking of individual cells using high-resolution PET imaging [37]. Although it is a straightforward approach, ex vivo labeling of T cells may cause cell toxicity because T cells are sensitive to radiation [38]. Therefore, cellular activities need to be examined after radiolabeling. In the present study, the cell viability was almost unaffected by ^64^Cu metabolic radiolabeling for up to 24 h, and after 48 h the labeled CTLs exhibited less DNA damage than CTLs exposed to X-ray radiation, which is comparable to the results recently reported using ^68^ Ga-mesoporous silica nanoparticle cell-labeling strategy [37]. Moreover, our labeling method did not affect the function of the CTLs, as determined using an enzyme-linked immunosorbent assay (ELISA) of IFN-γ. Notably, the glycan engineering method used in the present study resulted in favorable retention of radionuclide on the cell surface (88.42 ± 9.15% and 81.31 ± 6.40% for 24 and 48 h, respectively; Fig. 3d), which is markedly higher than that of the mesoporous silica nanoparticle method (less than 60% at 24 h) [37]. The high retention of radionuclide on the cell surface guaranteed the accurate detection of cells in vivo by PET imaging.

PET imaging studies revealed the specific targeting of ^64^Cu-labeled OVA-CTLs in the tumors (Fig. 3f), which was confirmed by ex vivo FACS analysis of the IRDye680-labeled OVA-CTLs (Fig. 2f). These results suggest that the PET imaging strategy could be used to monitor the in vivo tumor targeting efficiency and predict the antitumor effect of CTLs*.* In a clinical setting, traditional imaging methods such as computed tomography (CT) and magnetic resonance imaging (MRI), which provide anatomical information, may be used to monitor tumor responses to T cell-based adoptive transfer therapy. However, simply monitoring tumor size is a poor indicator of response because efficacious therapy may increase the number of immune cells that infiltrate the tumor, thereby increasing its diameter [39]. Functional imaging using ^18^F-FDG—the most widely used clinical PET tracer—may overcome some of the limitations of anatomical imaging. However, because it exhibits high uptake in both tumor-infiltrating T cells and cancer cells [40], it has limitations in differentiating between tumor progression and T cell infiltration-induced pseudoprogression. In contrast, direct PET imaging of radiolabeled CTLs using the method reported herein allows the direct quantification of the CTLs that have infiltrated the tumor in vivo*.* This would enable to predict the antitumor efficacy and determine the off-target organs of the transplanted T cells.

As a proof-of-concept to evaluate whether PET imaging using ^64^Cu-labeled OVA-CTLs could be used to predict the early-stage treatment efficacy and to select drugs for adjuvant cell-based therapies, we determined the tumor uptake changes in ^64^Cu-labeled OVA-CTLs after treatment with the FAK-inhibiting nanodrug (PLGA-FAKi). PET imaging revealed the enhanced infiltration of OVA-CTLs following PLGA-FAKi treatment (Fig. 4f), suggesting that PLGA-FAKi increases the tumor infiltration of OVA-CTLs. Mechanistic investigation revealed that PLGA-FAKi treatment reduced collagen formation, downregulated the expression of FAP, and remodeled the tumor microenvironment by increasing CD8^+^ T cell infiltration and reducing the number of Treg cells and M2 macrophages, which is consistent with those of the other study on FAK inhibition [15]. The improved antitumor effect of the adoptive transfer of OVA-CTLs combined with PLGA-FAKi treatment confirmed the value of PET imaging for predicting the combination therapy efficacy. Because our proposed metabolic radiolabeling strategy could be done easily within 24 h and has potential for clinical translation, it also offers opportunities for the noninvasive PET monitoring of other cell-based adoptive therapies.

Notably, ex vivo radiolabeling of T cells followed by in vivo PET imaging as described in this study has certain limitations, i.e., it is difficult to differentiate between alive and dead cells, and to track proliferated cells [31]. In addition, long-term imaging is challenging because the label becomes diluted as T cells multiply [18, 31]. Therefore, PET imaging of radiolabeled T cells can only provide a quick insight into the early-stage migration of T cells after transplantation. Combination strategies with anatomical imaging of changes in tumor sizes, PET imaging of key biomarkers associated with T cell activation, and serum examination of cytokines may be needed to provide the complementary information for mapping the whole profile of transferred T cells in vivo.

## Conclusion

We showed that CTLs can be metabolically radiolabeled to enable PET imaging of their rapid migration and tumor-targeting efficiency in vivo. This strategy may be used clinically for direct cell labeling that offers opportunities for the noninvasive monitoring of cell-based adoptive therapies and guiding the selection of adjuvant therapeutics.

## Materials and methods

### Reagents

All commercially obtained chemicals were of analytical grade. IRDye680-dibenzo-cyclooctyne (DBCO), DBCO-PEG_4_-NH_2_, and *N*-azidoacetylmannosamine (Ac_4_ManNAz) were purchased from Click Chemistry Tools (Scottsdale, AZ). The chelator *S*-2-(4-Isothiocyanatobenzyl)-1,4,7-triazacyclononane-1,4,7-triacetic acid (*p*-SCN-Bn-NOTA) was purchased from Macrocyclics (Dallas, TX). Female C57BL/6 mice (5 weeks of age) were purchased from Department of Laboratory Animal Science of Peking University (Beijing, China). The dye-labeled antibodies for flow cytometric analysis were purchased from eBioscience (San Diego, CA), unless otherwise noted.

### Cell culture and animal models

The chicken ovalbumin (OVA)-transfected mouse melanoma cell line (B16-OVA) was kindly provided by Dr. Jing Huang at Peking University. The B16-OVA cells were grown in Dulbecco’s modified eagle medium (Invitrogen, Carlsbad, CA) supplemented with 10% FBS at 37 °C in a humidified atmosphere comprising 5% CO_2_ and were routinely screened for mycoplasma (Hoechst stain and PCR).

All animal experiments were performed by following the protocol approved by the institutional animal care and use committee at Peking University. The right hind legs of female C57BL/6 mice were subcutaneously injected with 1 × 10^6^ B16-OVA cells to establish a subcutaneous B16-OVA tumor mouse model. The growth of tumors was measured using a caliper, and the volume was calculated using the following formula: tumor volume = length × (width^2^)/2.

### Separation and culture of CTLs

OVA-specific T lymphocytes were isolated from the spleens of female C57BL/6 mice after four intraperitoneal injections of OVA (Bioss, Beijing, China) every 5 days. Each mouse was intraperitoneally injected with 25 μg of OVA in 200 μL of PBS and 100 μL of alum adjuvant. Mice treated with only 200 μL PBS and 100 μL of alum adjuvant were used as a vehicle control. The mouse spleens were harvested after immunization and ground in a pestle with PBS. After passing the resulting tissue through a 70-μm cell strainer, lymphocytes were separated by density gradient centrifugation according to the standard protocol. The lymphocytes were then activated by anti-CD3 and anti-CD28 antibodies (BD Biosciences), and expanded with interleukin-2 (IL-2) to generate OVA-specific cytotoxic T lymphocytes (OVA-CTLs) or control cytotoxic T lymphocytes (control-CTLs) [41]. The CTLs were cultured in RPMI-1640 medium (Invitrogen, Carlsbad, CA) containing 10% FBS, IL-2 (1500 IU/mL) (Genscript, Nanjing, China), and anti-CD3 antibodies (50 ng/mL; eBioscience) at 37 °C in a humidified atmosphere comprising 5% CO_2_.

### In vivo adoptive CTL therapy

B16-OVA tumor-bearing C57BL/6 mice with tumors of a uniform size (50–100 mm^3^) were divided into three groups (n = 5–6/group): a PBS-treated group, an OVA-CTL-treated group, and a control-CTL-treated group. Each mouse was injected with PBS or 5 × 10^5^ CTLs via its tail vein every day for 4 days. Tumor size and body weight were measured every other day.

### Metabolic labeling of CTLs with IRDye680

CTLs isolated from C57BL/6 mice were cultured in RPMI-1640 medium containing Ac_4_ManNAz (50 μg/mL) for 24 h. After washing, the cells were labeled with IRDye680-DBCO (0.25 μg/10^6^ cells) for 1 h. They were then washed three times in PBS, and analyzed with an LSRII flow cytometer (Becton Dickinson, Germany).

### ***Metabolic radiolabeling of the CTLs with ***^***64***^***Cu***

The CTLs were labeled with ^64^Cu using the metabolic strategy of incorporating azide (–N_3_) on the cell surface, followed by click chemistry conjugation of –N_3_ with ^64^Cu-NOTA-DBCO (Fig. 1). For the preparation of ^64^Cu-NOTA-DBCO, DBCO-PEG_4_-NH_2_ was first mixed with *p*-SCN-Bn-NOTA in a 1:10 ratio in sodium bicarbonate buffer (pH 8.5) at room temperature for 2 h. NOTA-conjugated DBCO-PEG_4_ (NOTA-DBCO) was isolated using semipreparative high-performance liquid chromatography (HPLC). The collected fractions were combined and lyophilized to yield the product. The NOTA-DBCO was obtained at 47% yield with > 95% HPLC purity. Matrix-assisted laser desorption/ionization (MALDI) time-of-light (TOF) mass spectrometry (MS): m/z 970.60 for [MH]^+^ (C_49_H_63_N_7_O_12_S, calculated molecular weight 970.14 Da).

For ^64^Cu radiolabeling, 25 μg of NOTA-DBCO was reacted with 222 MBq of ^64^CuCl_2_ in 300 μL of 0.1 M sodium acetate buffer (pH 5.5) at 37 °C for 30 min with constant shaking. The ^64^Cu-NOTA-DBCO was then purified using HPLC. The desired product (indicated by the radioactive peak) was collected and rotary evaporated to remove the solvent. The products were then formulated in PBS and passed through a 0.22-μm Millipore filter. The radiolabeling was performed with 90% decay-corrected yield for ^64^Cu-NOTA-DBCO. The in vitro stability of ^64^Cu-NOTA-DBCO was determined using instant thin layer chromatography after incubation in PBS or FBS for up to 24 h. The in vitro binding specificity of the Ac_4_ManNAz-incubated cells with regard to ^64^Cu-NOTA-DBCO was determined via a cell binding assay. Briefly, ^64^Cu-NOTA-DBCO (370 kBq) was added to 12-well plates containing Ac_4_ManNAz-incubated B16-OVA tumor cells. After incubation for 1 h, the plates were washed with PBS and the cells were collected. The cell-associated radioactivity was then measured using a γ-counter (Packard, Meriden, CT). Each experiment was performed twice using four samples.

^64^Cu-CTLs were produced by culturing Ac_4_ManNAz-treated CTLs with ^64^Cu-NOTA-DBCO. Briefly, CTLs were cultured in RPMI-1640 medium containing Ac_4_ManNAz (50 μg/mL) for 24 h. Then, the Ac_4_ManNAz-incubated CTLs were labeled with ^64^Cu-NOTA-DBCO for 1 h and washed three times with PBS.

### In vitro characterizations of the ^64^Cu-CTLs

A cell viability assay was performed to determine the effects of ^64^Cu radiolabeling on the growth of CTLs. Briefly, ^64^Cu-CTLs (5 × 10^3^/well) or unlabeled CTLs (5 × 10^3^/well) were seeded into 96-well plates and allowed to grow for 1, 4, 8, and 24 h, and the cell viability at each time-point was determined using Cell Counting Kit-8 (Dojindo Laboratories, Kumamoto, Japan).

The ^64^Cu efflux from ^64^Cu-CTLs was evaluated by incubating them in the culture medium at 37 °C in a humidified atmosphere comprising 5% CO_2_. After incubation for 1, 4, 8, 24, or 48 h, the cell culture medium was collected and the radioactivity in the medium was measured using a γ-counter. The percentage of ^64^Cu retained on the CTLs was then calculated as follows: percentage of ^64^Cu retention (%) = [1 − (radioactivity in the medium/total radioactivity)] × 100.

The damage caused to the DNA of the CTLs by ^64^Cu radiolabeling was determined using γH2AX immunofluorescence staining. Briefly, ^64^Cu-CTLs or unlabeled CTLs were cultured for 3, 8, 24, or 48 h. At each time point, the CTLs were fixed using 4% paraformaldehyde and washed with PBS. The cells were then incubated overnight with anti-phosphorylated-histone H2AX (Ser 139) antibodies (1:100; Merck Millipore, Bedford, MA) at 4 °C, treated with a DyLight 549-labeled secondary antibody (Earthox, Millbrae, CA), and examined under a Leica TCS-NT confocal microscope (Leica, Wetzlar, Germany). As a positive control, the CTLs were irradiated with 3 or 6 Gy using an X-ray irradiator (RS2000 PRO, 160 kV, 25 mA; Rad Source Technologies, Suwanee, GA). CTLs were then fixed and stained using the same protocol described above. The mean fluorescence intensity of γH2AX for each CTL was quantified using ImageJ software (NIH, Bethesda, MD).

### Preparation and characterization of PLGA-FAKi

PF-562271 (Selleck Chemicals, Houston, TX), which is a FAK inhibitor (FAKi), was dissolved in dimethyl sulfoxide and added to a 10 mg/mL acetone solution of a PEG-grafted poly(lactic-co-glycolic acid) (PLGA) co-polymer (PEG-PLGA; 50:50 (w/w); Mw ~ 5000:10,000 Da; Xi’an ruixi Biological Technology Co., Ltd, Xi’an City, China). The ratio of PF-562271 to PLGA polymer was 1:67 (w/w). Then, 1 mL of the reaction mixture was added dropwise to 5 mL of water. After stirring for 1 h and standing for 12 h, the PF-562271-containing PLGA nanoparticles (PLGA-FAKi) were purified using centrifugation at 30,000*g* for 5 min, and washed with PBS. The particle size distribution and zeta potential of the PLGA-FAKi were measured using dynamic light scattering (Brookhaven Instruments, Holtsville, NY), and the morphology of the PLGA-FAKi was characterized using a JEM-1400 transmission electron microscope (JEOL, Japan). The release ratio of PF-562271 was determined using HPLC with an ultraviolet–visible detector at 210 nm.

### Combination of PLGA-FAKi and adoptive CTL therapy

The B16-OVA tumor-bearing mice were divided into 6 groups (n = 7/group): (1) PBS control, (2) PLGA alone, (3) PLGA-FAKi alone, (4) OVA-CTLs, (5) OVA-CTLs plus PLGA, and (6) OVA-CTLs plus PLGA-FAKi. In the PLGA and PLGA-FAKi groups, the mice were injected with PLGA or PLGA-FAKi (50 μg of FAKi equivalent) on day 0 via their tail veins. In the OVA-CTLs groups, the mice were injected with 5 × 10^5^ OVA-CTLs every day for 4 days (from day 0 to day 3) via their tail veins. The tumor growth and body weights of each mouse were monitored every other day.

When the treatments were terminated (day 14), 5 mice from each group were euthanized and the tumors were harvested. The tumors were immediately frozen in OCT medium and cut into 5-μm-thick slices for immunofluorescence staining of Ki67.

### ***PET imaging of ***^***64***^***Cu-labeled CTLs***

Each B16-OVA tumor-bearing mouse (n = 4/group) was intravenously injected with 3.7 MBq of ^64^Cu-labeled CTLs isolated from mice immunized with or without OVA (OVA-CTLs or control-CTLs), and 10-min static PET scans were obtained at 4, 12, 24, and 36 h postinjection using a small-animal PET/CT scanner (Siemens Medical Solutions). The PET images were analyzed, and the region-of-interest-derived percentage injected dose per gram of tissue (%ID/g) was calculated. In a separate experiment, B16-OVA tumor-bearing mice (n = 4/group) were administered with PLGA or PLGA-FAKi (50 μg of FAKi equivalent) on day 0 via their tail veins. On day 5, the mice were intravenously injected with 3.7 MBq of ^64^Cu-labeled OVA-CTLs, and 10-min static PET scans were obtained at 24 and 32 h postinjection.

### SPECT imaging of HSA in vivo

To evaluate the role of PLGA-FAKi in tumor vasculature perfusion, SPECT imaging was performed using ^99m^Tc-HSA. To prepare the ^99m^Tc-HSA, HSA was first conjugated with the chelator 6-hydrazinonicotinyl, and then labeled with Na^99m^TcO_4_ using a previously described protocol [42]. For SPECT imaging, each mouse (n = 3–4/group) was intravenously injected with 148 MBq ^99m^Tc-HSA and SPECT/CT imaging was performed at 12 and 24 h postinjection using a NanoScan SPECT/CT Imaging System (Mediso, Budapest, Hungary).

### Flow cytometry analysis

IRDye680 labeled control-OVAs or OVA-CTLs were intravenously injected into B16-OVA tumor-bearing mice and the tumors were harvested at 24 h postinjection. Each tumor was digested to obtain single-cell suspensions, as previously described [43]. The single-cell suspensions were stained with fluorescein isothiocyanate (FITC)-conjugated anti-CD3 and phycoerythrin (PE)-conjugated anti-CD8 antibodies, and subsequently sorted using an LSRII flow cytometer.

In a separate experiment, B16-OVA tumor-bearing mice were intravenously injected with PLGA or PLGA-FAKi (50 μg of FAKi equivalent). The tumors were harvested at 36 h postinjection and digested as mentioned earlier. After PLGA-FAKi therapy, single-cell suspensions were stained with anti-CD3 (FITC), anti-CD8 (PE), anti-CD4 (allophycocyanin (APC)), anti-FoxP3 (PE), anti-CD206 (FITC), and anti-F4/80 (PE) for the flow cytometric analysis of CD4^+^ T cells, CD8^+^ T cells, regulatory T cells (Treg cells), and tumor-associated macrophages, respectively.

### Sirius red staining and immunofluorescence staining

B16-OVA tumors were fixed with 10% formalin, embedded in paraffin, and cut into slices for staining with Sirius Red, as previously described [44].

For immunofluorescence staining, frozen B16-OVA tumor sections were fixed with ice-cold acetone, rinsed with PBS, and blocked with 10% FBS for 1 h at room temperature. The tumor slices were incubated with rabbit anti-mouse Ki67 antibody (Millipore) or rabbit anti-mouse fibroblast activation protein (FAP)-α antibody (Millipore) for 1 h at room temperature, then visualized with dye-conjugated secondary antibodies (Earthox, Millbrae, CA) under a Leica TCS-NT confocal microscope. The tumor proliferation index was calculated as the percentage of Ki67-positive nuclei among the total number of nuclei, and the mean fluorescence intensity of FAP-α was quantified using the ImageJ software (NIH).

### Statistical analysis

Quantitative data are expressed as mean ± standard deviation (SD). Two-sample comparisons were analyzed using an unpaired two-tailed Student *t* test. For multi-groups statistical analysis, one-way analysis of variance (ANOVA) with a post-hoc Tukey test was performed. Tumor growth curves over time were compared by two-way ANOVA. Time-to-survival was expressed using Kaplan–Meier curves, and groups were compared using log-rank tests. *P* values less than 0.05 were considered statistically significant.

## Supplementary Information

**Additional file 1. Additional materials and methods and additional Figures S1–S5.**

## Data Availability

The datasets used and/or analyzed during the current study are available from the corresponding author (Z. L.) on reasonable request.

## References

[1] Rosenberg SA, Restifo NP (2015). Adoptive cell transfer as personalized immunotherapy for human cancer. Science.

[2] Restifo NP, Dudley ME, Rosenberg SA (2012). Adoptive immunotherapy for cancer: harnessing the T cell response. Nat Rev Immunol.

[3] Kosti P, Maher J, Arnold JN (2018). Perspectives on chimeric antigen receptor T-cell immunotherapy for solid tumors. Front Immunol.

[4] Mirzaei HR, Rodriguez A, Shepphird J, Brown CE, Badie B (2017). Chimeric antigen receptors T cell therapy in solid tumor: challenges and clinical applications. Front Immunol.

[5] Majzner RG, Mackall CL (2018). Tumor antigen escape from CAR T-cell therapy. Cancer Discov.

[6] Roybal KT, Rupp LJ, Morsut L, Walker WJ, McNally KA, Park JS (2016). Precision tumor recognition by T cells with combinatorial antigen-sensing circuits. Cell.

[7] Rabinovich BA, Ye Y, Etto T, Chen JQ, Levitsky HI, Overwijk WW (2008). Visualizing fewer than 10 mouse T cells with an enhanced firefly luciferase in immunocompetent mouse models of cancer. Proc Natl Acad Sci U S A.

[8] Gajewski TF, Schreiber H, Fu YX (2013). Innate and adaptive immune cells in the tumor microenvironment. Nat Immunol.

[9] Junttila MR, de Sauvage FJ (2013). Influence of tumour micro-environment heterogeneity on therapeutic response. Nature.

[10] Sulzmaier FJ, Jean C, Schlaepfer DD (2014). FAK in cancer: mechanistic findings and clinical applications. Nat Rev Cancer.

[11] Zhai J, Lin H, Nie Z, Wu J, Canete-Soler R, Schlaepfer WW (2003). Direct interaction of focal adhesion kinase with p190RhoGEF. J Biol Chem.

[12] Liu Y, Loijens JC, Martin KH, Karginov AV, Parsons JT (2002). The association of ASAP1, an ADP ribosylation factor-GTPase activating protein, with focal adhesion kinase contributes to the process of focal adhesion assembly. Mol Biol Cell.

[13] Hildebrand JD, Taylor JM, Parsons JT (1996). An SH3 domain-containing GTPase-activating protein for Rho and Cdc42 associates with focal adhesion kinase. Mol Cell Biol.

[14] Stokes JB, Adair SJ, Slack-Davis JK, Walters DM, Tilghman RW, Hershey ED (2011). Inhibition of focal adhesion kinase by PF-562,271 inhibits the growth and metastasis of pancreatic cancer concomitant with altering the tumor microenvironment. Mol Cancer Ther.

[15] Jiang H, Hegde S, Knolhoff BL, Zhu Y, Herndon JM, Meyer MA (2016). Targeting focal adhesion kinase renders pancreatic cancers responsive to checkpoint immunotherapy. Nat Med.

[16] Roberts WG, Ung E, Whalen P, Cooper B, Hulford C, Autry C (2008). Antitumor activity and pharmacology of a selective focal adhesion kinase inhibitor, PF-562,271. Cancer Res.

[17] Krebs S, Ahad A, Carter LM, Eyquem J, Brand C, Bell M (2018). Antibody with infinite affinity for in vivo tracking of genetically engineered lymphocytes. J Nucl Med.

[18] Krebs S, Ponomarev V, Slovin S, Schöder H (2019). Imaging of CAR T-cells in cancer patients: paving the way to treatment monitoring and outcome prediction. J Nucl Med.

[19] James ML, Gambhir SS (2012). A molecular imaging primer: modalities, imaging agents, and applications. Physiol Rev.

[20] Agatemor C, Buettner MJ, Ariss R, Muthiah K, Saeui CT, Yarema KJ (2019). Exploiting metabolic glycoengineering to advance healthcare. Nat Rev Chem.

[21] Prescher JA, Bertozzi CR (2005). Chemistry in living systems. Nat Chem Biol.

[22] Kolb HC, Finn MG, Sharpless KB (2001). Click chemistry: diverse chemical function from a few good reactions. Angew Chem Int Ed Engl.

[23] Chang PV, Prescher JA, Sletten EM, Baskin JM, Miller IA, Agard NJ (2010). Copper-free click chemistry in living animals. Proc Natl Acad Sci U S A.

[24] St Paul M, Ohashi PS (2020). The roles of CD8+ T cell subsets in antitumor immunity. Trends Cell Biol.

[25] Guedan S, Ruella M, June CH (2019). Emerging cellular therapies for cancer. Annu Rev Immunol.

[26] Ashmore-Harris C, Iafrate M, Saleem A, Fruhwirth GO (2020). Non-invasive reporter gene imaging of cell therapies, including T cells and stem cells. Mol Ther.

[27] Keu KV, Witney TH, Yaghoubi S, Rosenberg J, Kurien A, Magnusson R (2017). Reporter gene imaging of targeted T cell immunotherapy in recurrent glioma. Sci Transl Med.

[28] Yaghoubi SS, Jensen MC, Satyamurthy N, Budhiraja S, Paik D, Czernin J (2009). Noninvasive detection of therapeutic cytolytic T cells with 18F-FHBG PET in a patient with glioma. Nat Clin Pract Oncol.

[29] Zhang H, Moroz MA, Serganova I, Ku T, Huang R, Vider J (2011). Imaging expression of the human somatostatin receptor subtype-2 reporter gene with 68Ga-DOTATOC. J Nucl Med.

[30] Castanares MA, Mukherjee A, Chowdhury WH, Liu M, Chen Y, Mease RC (2014). Evaluation of prostate-specific membrane antigen as an imaging reporter. J Nucl Med.

[31] Liu Z, Li Z (2014). Molecular imaging in tracking tumor-specific cytotoxic T lymphocytes (CTLs). Theranostics.

[32] Berglund D, Karlsson M, Palanisamy S, Carlsson B, Korsgren O, Eriksson O (2013). Imaging the in vivo fate of human T cells following transplantation in immunoincompetent mice—implications for clinical cell therapy trials. Transpl Immunol.

[33] Pittet MJ, Grimm J, Berger CR, Tamura T, Wojtkiewicz G, Nahrendorf M (2007). In vivo imaging of T cell delivery to tumors after adoptive transfer therapy. Proc Natl Acad Sci U S A.

[34] Weist MR, Starr R, Aguilar B, Chea J, Miles JK, Poku E (2018). PET of adoptively transferred chimeric antigen receptor T cells with 89Zr-Oxine. J Nucl Med.

[35] Adonai N, Adonai N, Nguyen KN, Walsh J, Iyer M, Toyokuni T (2002). Ex vivo cell labeling with 64Cu-pyruvaldehyde-bis(N4-methylthiosemicarbazone) for imaging cell trafficking in mice with positron-emission tomography. Proc Natl Acad Sci U S A.

[36] Griessinger CM, Maurer A, Kesenheimer C, Kehlbach R, Reischl G, Ehrlichmann W (2015). 64Cu antibody-targeting of the T-cell receptor and subsequent internalization enables in vivo tracking of lymphocytes by PET. Proc Natl Acad Sci U S A.

[37] Jung KO, Kim TJ, Yu JH, Rhee S, Zhao W, Ha B (2020). Whole-body tracking of single cells via positron emission tomography. Nat Biomed Eng.

[38] Minn I, Rowe SP, Pomper MG (2019). Enhancing CAR T-cell therapy through cellular imaging and radiotherapy. Lancet Oncol.

[39] Seo JW, Tavare R, Mahakian LM, Silvestrini MT, Tam S, Ingham ES (2018). CD8+ T-cell density imaging with 64Cu-labeled cys-diabody informs immunotherapy protocols. Clin Cancer Res.

[40] Laing RE, Nair-Gill E, Witte ON, Radu CG (2010). Visualizing cancer and immune cell function with metabolic positron emission tomography. Curr Opin Genet Dev.

[41] Ruan S, Lin M, Zhu Y, Lum L, Thakur A, Jin R (2020). Integrin β4-targeted cancer immunotherapies inhibit tumor growth and decrease metastasis. Cancer Res.

[42] Zhao Y, Zhang C, Gao L, Yu X, Lai J, Lu D (2017). Chemotherapy-induced macrophage infiltration into tumors enhances nanographene-based photodynamic therapy. Cancer Res.

[43] Zhang C, Gao L, Cai Y, Liu H, Gao D, Lai J (2016). Inhibition of tumor growth and metastasis by photoimmunotherapy targeting tumor-associated macrophage in a sorafenib-resistant tumor model. Biomaterials.

[44] Yu X, Wu Y, Liu H, Gao L, Sun X, Zhang C (2016). Small-animal SPECT/CT of the progression and recovery of rat liver fibrosis by using an integrin αvβ3-targeting radiotracer. Radiology.

