# Supplementary material for: Metabolic radiolabeling and in vivo PET imaging of cytotoxic T lymphocytes to guide combination adoptive cell transfer cancer therapy
Source: J Nanobiotechnology. 2021 Jun 10;19:175. doi: 10.1186/s12951-021-00924-2 (PMC8194184; doi:10.1186/s12951-021-00924-2)
Supplement: Supplementary file 1 — Additional file 1. Additional materials and methods and additional Figures S1–S5. [file 12951_2021_924_MOESM1_ESM.docx]

**Additional Information for**

**Metabolic radiolabeling and in vivo PET imaging of cytotoxic T lymphocytes to guide combination adoptive cell transfer cancer therapy**

Dehua Lu^1^, Yanpu Wang^1^, Ting Zhang^1^, Feng Wang^2^, Kui Li^1^, Shixin Zhou^3^, Hua Zhu^2,4^*, Zhi Yang^2,4^*, Zhaofei Liu^1,4^*

^1^Medical Isotopes Research Center and Department of Radiation Medicine, School of Basic Medical Sciences, Peking University Health Science Center, Beijing 100191, China

^2^Key Laboratory of Carcinogenesis and Translational Research (Ministry of Education/Beijing), Department of Nuclear Medicine, Peking University Cancer Hospital & Institute, Beijing 100142, China

^3^Department of Cell Biology, School of Basic Medical Sciences, Peking University Health Science Center, Beijing 100191, China

^4^NMPA Key Laboratory for Research and Evaluation of Radiopharmaceuticals (National Medical Products Administration), Peking University Cancer Hospital & Institute, Beijing 100142, China

***Corresponding Authors:** Zhaofei Liu, Medical Isotopes Research Center, Peking University Health Science Center, Beijing 100191, China. E-mail: liuzf@bjmu.edu.cn; Hua Zhu, Department of Nuclear Medicine, Peking University Cancer Hospital & Institute, Beijing, 100142, China. Email: zhuhuananjing@163.com; Zhi Yang, Department of Nuclear Medicine, Peking University Cancer Hospital & Institute, Beijing, 100142, China. Email: pekyz@163.com

**Additional Materials and Methods**

**In vitro cell lysis of cytotoxic T lymphocytes (CTLs)**

To determine the interaction between CTLs and tumor cells, CTLs and B16-OVA cells were pre-labeled with carboxyl dimethyl fluorescein diacetate (CMFDA) and CM-Dil (Invitrogen, Carlsbad, CA), respectively. B16-OVA cells grown on 35-mm MatTek glass-bottomed culture dishes were incubated with control CTLs or ovalbumin-specific CTLs (OVA-CTLs). Cell interactions were dynamically visualized under a Leica TCS-NT confocal microscope (Leica, Wetzlar, Germany).

To determine the cytotoxic effect of CTLs on tumor cells, OVA-CTLs or control-CTLs were co-cultured with B16-OVA tumor cells in six-well plates at a ratio of 1:2 for 24 h. After washing with PBS, the cells were stained with phosphatidyl inositol (PI) (Dojindo Laboratories, Kumamoto, Japan) for 5 min, and then analyzed using flow cytometry.

**Detection of IFN-γ secretion by the CTLs**

To investigate the effects of Ac_4_ManNAz incubation or DBCO reaction on IFN-γ secretion by the CTLs, control-CTLs, OVA-CTLs, OVA-CTLs pretreated with Ac_4_ManNAz, OVA-CTLs pretreated with DBCO-PEG_4_-NHS, or OVA-CTLs pretreated with Ac_4_ManNAz plus DBCO-PEG_4_-NHS were co-cultured at a ratio of 1:2 with B16-OVA tumor cells for 24 h in 6-well plates. The supernatant of the cultured cells was then collected and IFN-γ levels were determined using an enzyme-linked immunosorbent assay (ELISA) kit (Invitrogen, Carlsbad, CA).

**Biodistribution of ^64^Cu-labeled CTLs**

B16-OVA tumor-bearing mice (n = 5/group) were intravenously injected with 0.74 MBq ^64^Cu-labeled CTLs or ^64^Cu-labeled OVA-CTLs. At 36 h postinjection, mice were euthanized and dissected. Blood, tumors, and major organs/tissues were harvested and weighed. Radioactivity in tissues and organs was measured using a γ-counter (Packard, Meriden, CT), and the results are presented as percentage injected dose per gram of tissue (%ID/g).

**In vivo optical** **imaging of human serum albumin (HSA)**

To evaluate the role of PLGA-FAKi in tumor vasculature perfusion, optical imaging was performed using IRDye800-labeled human serum albumin (Dye-HSA). The Dye-HSA was prepared by mixing HSA with IRDye800-N-hydroxysuccinimide (LI-COR, Inc., Lincoln, NE) at a molar ratio of 1: 10 in sodium bicarbonate solution (pH 8.4). After a 2 h reaction at 4°C, Dye-HSA was purified using a PD-10 desalting column (GE Healthcare, Piscataway, NJ). For optical imaging, each B16-OVA tumor-bearing mouse (n = 3–4/group) was injected with Dye-HSA (2 nmol) via its tail vein, and optical imaging was performed at 24 and 48 h postinjection using an IVIS small-animal imaging system (Xenogen, Alameda, CA).


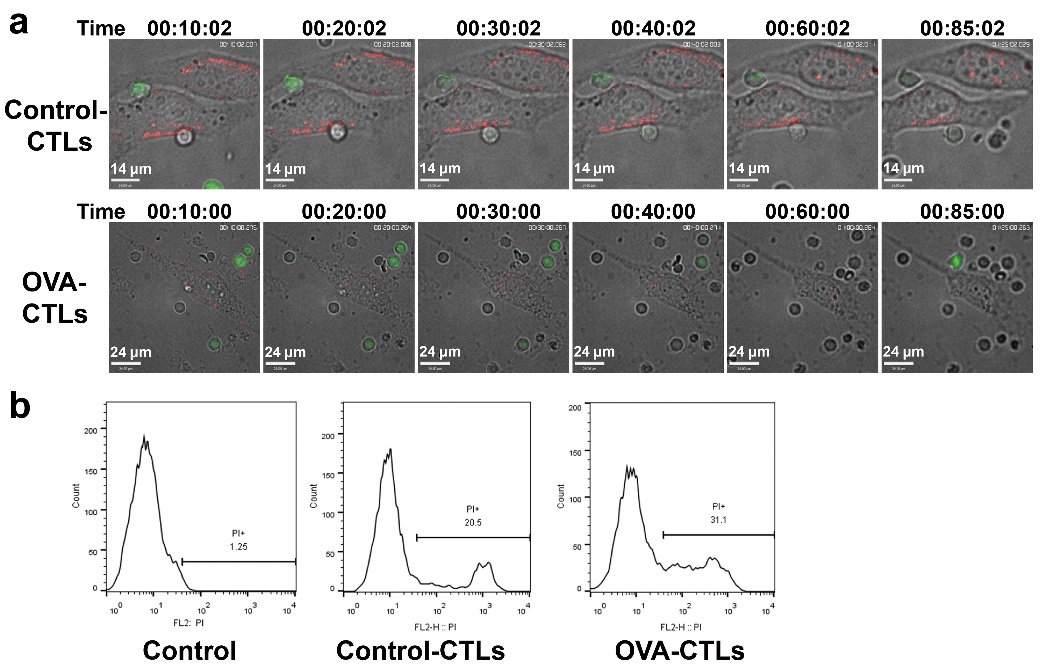


**Figure S1.** *In vitro* tumor cell lysis of OVA-specific cytotoxic T lymphocytes (OVA-CTLs). (**a**) Dynamic confocal fluorescence images of B16-OVA cells (CM-Dil; red) co-cultured with control-CTLs (CMFDA; green) or OVA-CTLs (CMFDA; green) at various times. (**b**) Flow cytometry analysis of B16-OVA cells treated with PBS (control), control-CTLs, or OVA-CTLs.

**
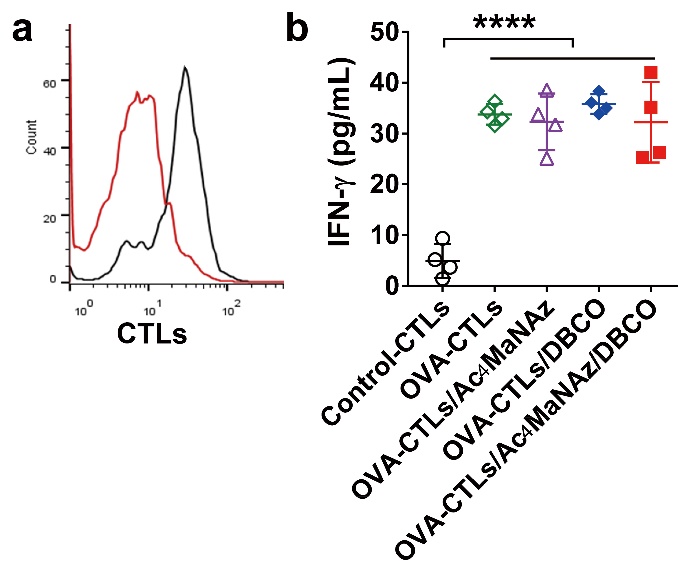
**

**Figure S2.** (**a**) Flow cytometry of cytotoxic T lymphocytes (CTLs) with (black line) or without (red line) pre-incubation with Ac_4_ManNAz, followed by staining with IRDye680-labeled DBCO. (**b**) Enzyme-linked immunosorbent assay (ELISA) of IFN-γ secretion by various CTLs after co-culture with B16-OVA tumor cells for 24 h. Control CTLs, OVA-CTLs, OVA-CTLs pretreated with Ac_4_ManNAz, OVA-CTLs pretreated with DBCO-PEG_4_-NHS, or OVA-CTLs pretreated with Ac_4_ManNAz plus DBCO-PEG_4_-NHS were examined. ****, *P* <0.0001.

**
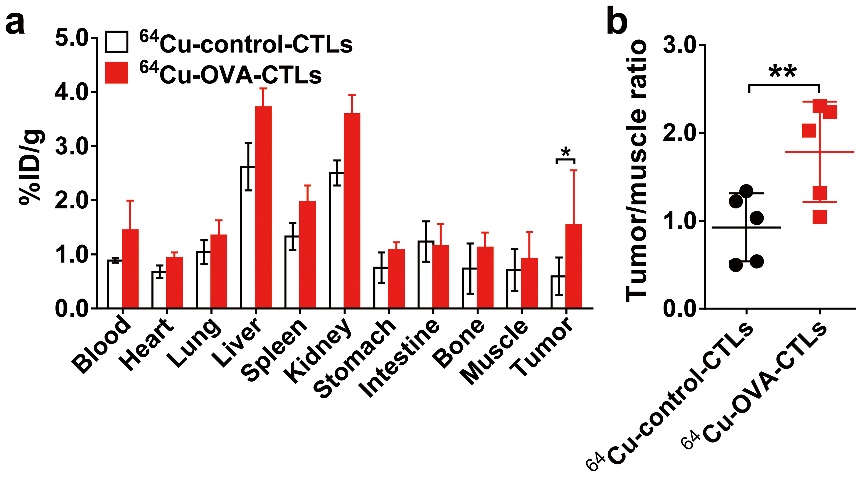
**

**Figure S3.** Biodistribution (**a**) and quantified tumor-to-muscle ratios (**b**) of B16-OVA tumor-bearing C57BL/6 mice after intravenous injection of ^64^Cu-labeled control cytotoxic T lymphocytes (control-CTLs) or ^64^Cu-labeled OVA-specific cytotoxic T lymphocytes (OVA-CTLs) at 36 h postinjection. Data are presented as mean ± SD. n = 5/group. *, *P* <0.05; **, *P* <0.01.

**
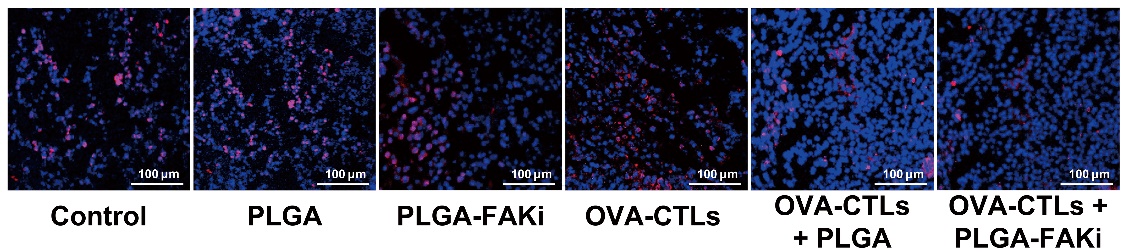
**

**Figure S4.** Ki67 immunofluorescence staining of B16-OVA tumors harvested from C57BL/6 mice after treatment with PBS (control), poly(lactic-co-glycolic) acid nanoparticles (PLGA), PLGA nanoparticle-encapsulated PF-562271 (PLGA-FAKi), OVA-specific cytotoxic T lymphocytes (OVA-CTLs), OVA-CTLs plus PLGA, or OVA-CTLs plus PLGA-FAKi.

**
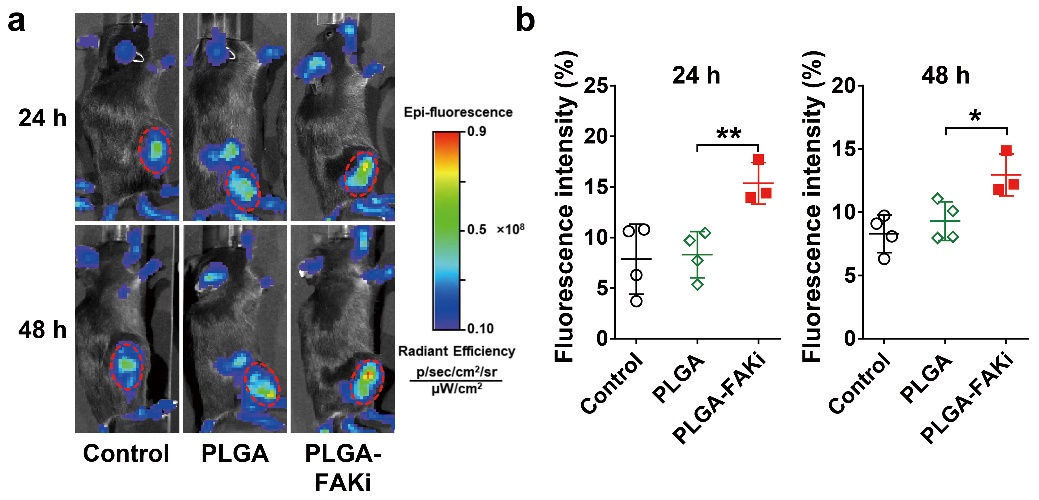
**

**Figure S5.** Optical images (**a**) and quantified tumor uptake (**b**) of IRDye800-labeled human serum albumin at 24 and 48 h postinjection in B16-OVA tumor-bearing C57BL/6 mice after treatment with PBS (control; n = 4), poly(lactic-co-glycolic) acid nanoparticles (PLGA) (n = 4), or PLGA nanoparticle-encapsulated PF-562271 (PLGA-FAKi) (n = 3). Tumors are indicated by dashed circles. Data are presented as mean ± SD. *, *P* <0.05; **, *P* <0.01.
